# Effect of Virtual Reality Simulation on Anatomy Learning Outcomes: A Systematic Review

**DOI:** 10.7759/cureus.81893

**Published:** 2025-04-08

**Authors:** Tochukwu S Odogwu, Esraa Abuelgassem Hagahmed Mohamed, Lailus Mishu, Izuchukwu Umahi

**Affiliations:** 1 Medical Education, Aston University, Birmingham, GBR; 2 Medicine, Southend University Hospital, Southend-on-Sea, GBR; 3 Internal Medicine, Mid and South Essex NHS Trust, Southend-on-Sea, GBR; 4 Medical Education, Mid and South Essex NHS Foundation Trust, Southend-on-Sea, GBR; 5 Pediatrics, Elizabeth Glaser Pediatric AIDS Foundation (EGPAF), Abuja, NGA

**Keywords:** anatomy education, learning outcomes in medical education, medical students, virtual reality in anatomy education, virtual reality in medical education

## Abstract

Anatomy education serves as a foundational pillar in medical training, equipping students with critical knowledge of human structure and function essential for clinical practice. Traditional pedagogical approaches, such as cadaveric dissection, anatomical models, and textbook-based learning, have been the cornerstone of medical education for decades. However, these methods face challenges, including limited accessibility (e.g., resource-intensive setups), ethical concerns (e.g., participant privacy), and difficulties in visualizing complex three-dimensional structures (e.g., dynamic systems). Recent advancements in immersive technologies, particularly virtual reality (VR), present a transformative opportunity to enhance anatomy education by offering interactive, scalable, and engaging learning experiences. This systematic review aimed to comprehensively evaluate the efficacy of VR simulation in improving medical students' learning outcomes in anatomy education, comparing its effectiveness to conventional teaching methodologies.

This systematic review was conducted in strict accordance with Preferred Reporting Items for Systematic Reviews and Meta-Analyses (PRISMA) and Cochrane guidelines to ensure methodological rigor. A comprehensive literature search was performed across six major medical databases, including PubMed, Cochrane Library and Web of Science, encompassing studies published between January 2019 and February 2024. The search strategy employed a combination of MeSH terms and keywords related to "virtual reality", "anatomy education", and "medical students". Study selection was guided by the Population, Intervention, Comparison, Outcome and Study (PICOS) framework, with inclusion criteria focusing on randomized controlled trials, quasi-experimental studies, and comparative analyses evaluating VR-based anatomy instruction for medical students. The Cochrane risk-of-bias tool was used to assess study quality and potential bias. Due to significant heterogeneity in study designs and outcome measures, a narrative synthesis approach was adopted to integrate findings thematically rather than through meta-analysis.

The review included 14 studies with a total of 961 participants, predominantly medical students across various training stages. Among the included studies, 71% demonstrated statistically significant improvements in anatomy learning outcomes with VR compared to traditional methods, including enhanced spatial understanding, knowledge retention, and learner engagement; 21% of the studies reported no significant difference in learning efficacy between VR and conventional approaches, while only 7% found VR simulation only partially effective, citing technical limitations and a steep learning curve as barriers. Subgroup analyses suggested that VR was particularly beneficial for visualizing complex anatomical regions (e.g., neuroanatomy, musculoskeletal structures) and for students with limited prior exposure to cadaveric dissection. However, variability in VR platform design, assessment tools and instructional integration underscored the need for standardized implementation frameworks.

The review generally indicated the benefits of VR simulation in anatomy education, but larger studies are needed to fully explore its potential in medical anatomy training. Future studies should rigorously evaluate VR implementation in anatomy education to provide more comprehensive evidence of its strengths and weaknesses for medical teaching.

## Introduction and background

Rationale and context of the study

The landscape of medical education, particularly in the domain of anatomy, has undergone significant transformations with the advent of innovative technologies. Among these, virtual reality (VR) has emerged as an important tool, offering new insights into educational methodologies and potentially enhancing learning outcomes for medical students.

Anatomy, a foundational pillar of medical education, requires a deep understanding of the complex spatial relationships and structures within the human body. Traditionally, this understanding has been facilitated through methods such as cadaver dissection, textbooks, and 2D imaging [1]. However, these methods have limitations, including the high costs associated with cadaver maintenance, ethical concerns, and the static nature of 2D representations that fail to fully convey the three-dimensional intricacies of human anatomy [2].

Virtual reality, characterized by its immersive and interactive environment, offers a dynamic and engaging learning platform. By simulating real-life scenarios and anatomical structures in a controlled, virtual space, VR allows students to visualize and manipulate anatomical parts in ways that were previously confined to cadaveric dissection [2-4]. This not only aids in overcoming the logistical and ethical constraints associated with traditional methods but also enhances the accessibility and repeatability of learning experiences.

Synthesis of the current literature

Recent studies have highlighted the potential of VR to improve the understanding of complex anatomical structures significantly. For instance, VR applications enable the visualization of anatomical layers as they would appear in real life, allowing for a more intuitive understanding of spatial relationships [3,5]. Moreover, the interactive component of VR has been shown to increase student engagement and motivation, potentially leading to better retention of information and higher satisfaction rates [6].

Comparative studies have also been conducted to evaluate the efficacy of VR against traditional learning methods. These studies often assess various outcomes, including examination scores, retention rates, and student preferences. While some research indicates that VR can lead to higher examination scores and better retention, the results are not uniformly positive across all studies [7]. These inconsistencies highlight the critical need for standardized implementation frameworks to optimize VR’s integration [8,9].

Furthermore, the implementation of VR in medical education raises questions about the required infrastructure, the training of educators, and the cost-effectiveness of such technology. While cost analyses fall beyond this review’s scope, existing studies suggest both VR and cadaveric methods present unique financial trade-offs that institutions must weigh against educational objectives. As VR technology continues to evolve, ongoing research is necessary to address these challenges and to refine VR applications to meet educational needs effectively as successful case studies demonstrate its potential when deployed systematically [10,11].

Aim of the research

This systematic review aims to comprehensively assess the impact of VR simulation on medical student learning outcomes in anatomy education. This review seeks to consolidate existing knowledge, critically evaluate the effectiveness of VR in comparison to traditional methods, and identify key themes and trends in the current literature. The following were the study objectives: (1) to assess the effectiveness of VR simulation relative to conventional methods, (2) to examine varied learning outcomes across both VR and traditional modalities, and (3) to evaluate differences in student engagement and satisfaction between approaches.

## Review

This systematic review was conducted in accordance with the guidelines outlined in the Cochrane Collaboration Handbook for Systematic Reviews [12], and adheres to the Preferred Reporting Items for Systematic Reviews and Meta-Analyses (PRISMA) framework to ensure methodological rigor and transparent reporting [13]. The primary research question guiding this review was "What is the comparative effectiveness of virtual reality simulation versus traditional methods in enhancing medical student learning outcomes within the context of anatomy education?" This question was designed to evaluate the efficacy of emerging virtual reality technologies relative to conventional pedagogical approaches in anatomy training.

Search strategy

A comprehensive search of six medical and educational databases yielded over 70,000 initial results. The Population, Intervention, Comparison, Outcome and Study (PICOS) framework [14] guided the development of inclusion and exclusion criteria (Table 1). After applying filters based on these criteria, 339 relevant studies were identified for further review. This systematic approach ensured a focused and manageable set of literature for analysis.

**Table 1:** Population, Intervention, Comparison, Outcome and Study (PICOS) framework showing the inclusion and exclusion criteria used for the review RCT: randomized controlled trials

Framework	Inclusion	Exclusion
Population	Medical students as the primary target audience	Studies involving healthcare professionals other than medical students
Intervention	Studies that employ virtual reality (VR) simulation as an educational tool for anatomy learning	Articles NOT focusing on virtual reality applications or unrelated to anatomy education
Comparison	Studies with appropriate control groups or comparative interventions (traditional methods, other educational technologies)	Studies WITHOUT controls or comparators
Outcome	Quantifiable measures of learning outcomes, including, but not limited to, examination scores, practical assessments, or objective structured clinical examinations (OSCEs); reports assessing specific learning outcomes such as knowledge acquisition, skill development, or performance improvement	Studies lacking specific assessments of anatomy learning; studies without quantifiable measures of learning outcomes
Study	Studies published between 2019 and 2024; English language articles or have English translation available; primary research studies with RCTs, experimental, quasi-experimental, or observational designs	Studies published before 2019; non-English language articles; reviews, editorials, letters, commentaries, case reports, and non-research articles

**Figure 1:**
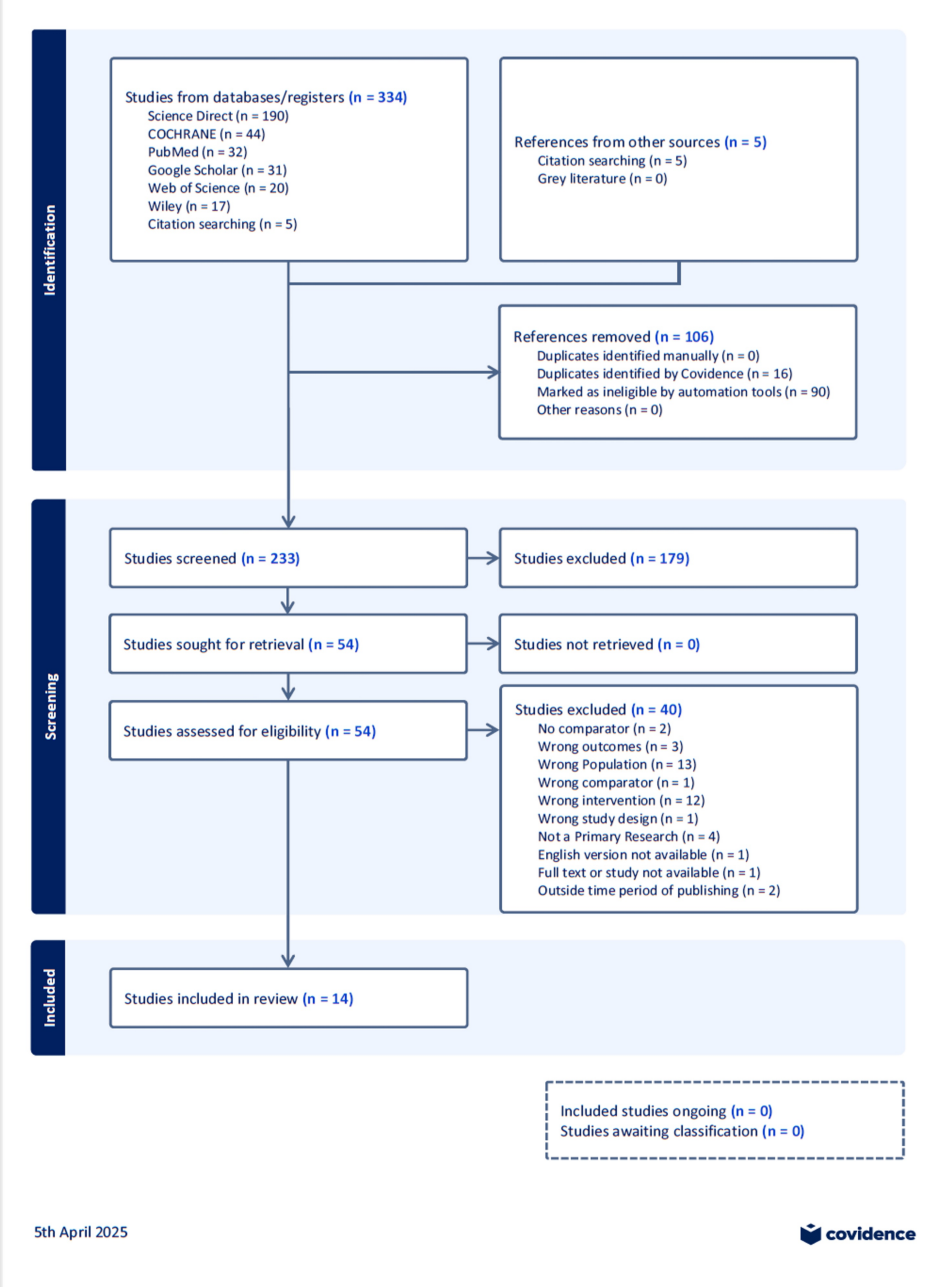
Preferred Reporting Items for Systematic Reviews and Meta-Analyses (PRISMA) flow diagram for the systematic review Covidence systematic review software, Veritas Health Innovation, Melbourne, Australia. Available at www.covidence.org.

Initially, the titles and abstracts of relevant articles were screened, followed by a review of the main texts. Articles were selected according to predefined criteria, and some were excluded with documented reasons for their removal. Ultimately, 14 papers were chosen for analysis to extract their general characteristics, interventions, and research findings, as detailed in Figure 1.

Database search was spread across six research publication sites with the results, as well as key words, summarized in Table 2.

**Table 2:** Database search results

	Database	Search string/key words	Initial hits	Filter 1: Publish date 2019-2024 (all fields), best match	Filter 2: Publish date 2019-2024 (all fields), best match medicine/medical education field related only	Filter 3: Publish date 2019-2024 (all fields), best match medicine/medical education field related, primary research only	Results after relevance screening
1	Google Scholar	virtual reality AND anatomy education AND medical student learning outcome	48000	17500	694	31	31
2	Wiley	virtual reality AND anatomy education AND medical student learning outcome	21233	7891	259	17	17
3	Web of Science	TS=(Virtual reality AND anatomy education AND medical student learning outcomes)	59	59	48	20	20
4	Science Direct	virtual reality AND anatomy education AND medical student learning outcome	1393	735	513	190	190
5	Cochrane Library	virtual reality AND anatomy education AND medical student learning outcome	44	44	44	44	44
6	PubMed	virtual reality AND anatomy education AND medical student learning outcome	111	88	52	32	32
							334
7		Citation search					5
	Total		70,840				339
	Total following removal of duplicates and application of inclusion/exclusion criteria					Final number of studies imported for screening and review	339

Data extraction and study quality assessment

Data were extracted according to the Cochrane Handbook guidelines [12], with a detailed extraction table available on requested. Key characteristics analyzed included author, year, location, study design, evaluation methods, effectiveness, and type of virtual reality used. After extraction, the data were cross-checked against the original texts for accuracy. The quality and risk of bias were assessed using the Cochrane Collaboration’s tool [15], with assessments summarized in Table 3.

**Table 3:** Overview of risk of bias in the selected studies D1: randomization process; D2: deviations from the intended interventions; D3: missing outcome data; D4: measurement of the outcome; D5: selection of the reported result; PPT: PowerPoint

Study ID	Experimental	Comparator	Weight	D1	D2	D3	D4	D5	Overall bias
Imai et al. [16]	Virtual reality (VR) images	Lecture using 3D images	1	Some concerns	Low risk	Low risk	Low risk	Some concerns	Some concerns
Tapiala et al. [17]	Virtual reality training (VRT)	Traditional training (TT) using textbooks and images	1	Some concerns	Some concerns	Low risk	Some concerns	Some concerns	Some concerns
Greuter et al. [18]	3D VR models	2D images (CT angiograms or MR angiograms)	1	Some concerns	Low risk	Low risk	Low risk	Some concerns	Some concerns
Kane et al. [19]	Virtual reality learning environment (VRLE)	2D images	1	Low risk	Some concerns	Low risk	Low risk	Low risk	Low risk
Yang et al. [20]	VR simulator	Educational video	1	Low risk	Some concerns	Low risk	Some concerns	Some concerns	Some concerns
Ryan et al. [21]	VRLE	PowerPoint tutorial	1	Low risk	Low risk	Low risk	High risk	Low risk	Some concerns
Emadzadeh et al. [22]	Virtual dissection table (VDT)	Textbooks of topographical anatomy	1	Low risk	Some concerns	Some concerns	High risk	Some concerns	High risk
Cabrera et al. [23]	Self-directed practice of VR and the instructor-led practice of VR	No VR	1	Some concerns	Some concerns	Low risk	High risk	Some concerns	High risk
Korniienko and Barchi [24]	VR	Textbook-based learning	1	Low risk	Some concerns	Low risk	High risk	Some concerns	High risk
Sattar et al. [25]	Virtual reality-based learning	Text-based and video-based learning methodologies	1	Some concerns	High risk	Low risk	High risk	High risk	High risk
Maresky et al. [26]	Immersive cardiac VR experience	Independent study without VR intervention	1	Low risk	Low risk	Low risk	Low risk	Some concerns	Low risk
Alharbi et al. [27]	3D-VR tools	Traditional teaching methods, such as plastinated models	1	Some concerns	Some concerns	High risk	High risk	Some concerns	High risk
Copson et al. [28]	Stereoscopic 3D (S3D), monoscopic 3D (M3D)	2D noninteractive version (PPT)	1	Low risk	Some concerns	High risk	High risk	Some concerns	High risk
Chen et al. [29]	VR group and the cadaver group	Atlas group	1	Low risk	Some concerns	Low risk	High risk	Some concerns	Some concerns

The studies reviewed showed a range of bias concerns, with some studies demonstrating low risk and others moderate to high risk. Common issues across studies included the lack of blinding of participants and outcome assessors, potential selective reporting, and concerns about the randomization process. These factors can introduce performance, detection, and selection biases, which can affect the validity of the study's conclusions.

Synthesis method

The synthesis process adhered to the Synthesis Without Meta-analysis (SWiM) reporting guideline [30]. The primary focus was on evaluating the impact of VR devices on knowledge and skill acquisition across various medical specialties, particularly in anatomical training. The synthesis grouped studies by medical specialty, acknowledging the unique requirements of each field.

Relevant data on knowledge and skills gained, along with specific VR technologies employed, were extracted from each study. Risk of bias assessments yielded mixed results, as detailed in Table 3. Given the heterogeneity of study designs and reported outcomes, all 14 included studies were given equal consideration in the synthesis.

The diversity in quantitative and qualitative reporting of knowledge and skills outcomes, coupled with the varied nature of the studies, precluded a formal meta-analysis. Instead, a narrative synthesis approach was adopted to comprehensively analyze and present the findings.

Results

Study Characteristics

The review encompasses 14 studies (Table 4) from diverse countries, predominantly published between 2019 and 2024, indicating a rapidly evolving field of VR in medical education. Methodologically, the studies prioritized robust evidence, with 64% utilizing randomized controlled trials (RCTs), complemented by observational and mixed-methods approaches for a comprehensive understanding.

**Table 4:** Final selected studies for the review

Study ID	Study title	Lead author	Year of publication	Country in which the study was conducted	Study design
Imai et al. [16]	Incorporation of virtual reality in the clinical training of medical students studying esophageal and mediastinal anatomy and surgery	Takeharu Imai	2022	Japan	Prospective observational Study
Tapiala et al. [17]	Impact of virtual reality training on mastoidectomy performance: a prospective randomised study	Jesse Tapiala	2023	Finland	Randomized controlled trial
Greuter et al. [18]	Randomized study comparing 3D virtual reality and conventional 2D on-screen teaching of cerebrovascular anatomy	Ladina Greuter	2021	Switzerland	Randomized controlled trial
Kane et al. [19]	A randomized control trial of a virtual reality learning environment in obstetric medical student teaching	Daniel Kane	2022	Ireland	Randomized controlled trial
Yang et al. [20]	Cognitive and motor skill competence are different: results from a prospective randomized trial using virtual reality simulator and educational video in laparoscopic cholecystectomy	Cui Yang	2022	Germany	Randomized controlled trial
Ryan et al. [21]	Virtual reality learning: a randomized controlled trial assessing medical student knowledge of fetal development	Grace Ryan	2022	Ireland	Randomized controlled trial
Emadzadeh et al. [22]	Virtual dissection: an educational technology to enrich medical students' learning environment in gastrointestinal anatomy course	Ali Emadzade	2023	Iran	Quasi-experimental
Cabrera et al. [23]	Assessing the effectiveness of teaching anatomy with virtual reality	Mildred V. López Cabrera	2019	Mexico	Quantitative and quasi-experimental design
Korniienko and Barchi [24]	Influence of virtual reality tools on human anatomy learning	Inokentii A. Korniienko	2020	Ukraine	Randomized controlled trial
Sattar et al. [25]	Motivating medical students using virtual reality based education	Mian Usman Sattar	2020	Pakistan	Randomized controlled trial
Maresky et al. [26]	Virtual reality and cardiac anatomy: exploring immersive three-dimensional cardiac imaging, a pilot study in undergraduate medical anatomy education	H. S. Maresky	2019	Canada	Prospective cohort study
Alharbi et al. [27]	Three-dimensional virtual reality as an innovative teaching and learning tool for human anatomy courses in medical education: a mixed methods study	Yasser Alharbi	2020	Saudi Arabia	Convergent mixed-methods design
Copson et al. [28]	Development of a virtual reality clinically oriented temporal bone anatomy module with randomised control study of three-dimensional display technology	Bridget Copson	2021	Australia	Randomized controlled trial
Chen et al. [29]	Can virtual reality improve traditional anatomy education programmes? A mixed-methods study on the use of a 3D skull model	Shi Chen	2020	China	Randomized controlled trial

Evaluation methods combined quantitative assessments (71%) with qualitative approaches (29%), offering a mixed view of VR's impact on learning. The most common evaluation strategy paired skill tests with questionnaires (21%), assessing both practical abilities and knowledge acquisition.

While primarily focused on medical students (79%), the studies also included dental students and neurosurgery residents, suggesting VR's potential in specialized medical fields. The interventions covered a broad spectrum of anatomical areas, with 71% targeting specific regions and 14% addressing surgical procedures like laparoscopy.

Control groups employed various traditional learning methods, including cadaveric dissection, textbooks, and static images, enabling clear comparisons with VR approaches. Statistical analyses relied on established tests such as t-tests and ANOVA to assess learning outcome differences.

The VR technology used varied across studies, with some not specifying the system (14%), reflecting ongoing exploration in the field. This diversity highlights researchers' efforts to identify the most effective VR systems for medical education (Table 5).

**Table 5:** General characteristics of included studies 2D: two-dimensional; 3D: three-dimensional; XR: extended reality; CE: complete edition; ANOVA: analysis of variance; HSD: honestly significant difference; ANCOVA: analysis of covariance

Characteristics of included studies (N=14)		
	Frequency (n)	%
Year of publication		
2019	2	14%
2020	4	29%
2021	2	14%
2022	4	29%
2023	2	14%
Country of publication		
Australia	1	7%
Canada	1	7%
China	1	7%
Finland	1	7%
Germany	1	7%
Iran	1	7%
Ireland	2	14%
Japan	1	7%
Mexico	1	7%
Pakistan	1	7%
Saudi Arabia	1	7%
Switzerland	1	7%
Ukraine	1	7%
Study design		
Convergent mixed-methods design	1	7%
Prospective cohort study	1	7%
Prospective observational study	1	7%
Quantitative and quasi-experimental design	1	7%
Quasi-experimental	1	7%
Randomized controlled trial	9	64%
Evaluation methods		
Evaluation forms, structured forms, performance metrics	1	7%
Pretest and posttests, follow-up test, focus group discussions	1	7%
Pretest and posttest	1	7%
Pretest and posttest questionnaires	1	7%
Pretest, posttest, and follow-up examination	1	7%
Questionnaire	1	7%
Questionnaires and quizzes	1	7%
Questionnaires and tests	1	7%
Skill test and questionnaire	3	21%
Test and questionnaire	2	14%
Written test and questionnaire	1	7%
Data analysis		
Quantitative	4	29%
Quantitative and qualitative	10	71%
Study population		
Medical and dental students	1	7%
Medical students	11	79%
Medical students and neurosurgery residents	1	7%
Medical students and anatomy students	1	7%
Medical/surgical discipline		
Aneurysms	1	7%
Basic anatomy	1	7%
Cardiac anatomy	2	14%
Cholecystectomy	1	7%
Embryology	1	7%
Esophageal and mediastinal anatomy	1	7%
Gastrointestinal anatomy	1	7%
Laparoscopic surgery	1	7%
Mastoid anatomy	1	7%
Not specified	1	7%
Obstetrics anatomy	1	7%
Skull anatomy	1	7%
Temporal bone anatomy	1	7%
Type of control		
2D images	2	14%
3D images	1	7%
Anatomy books	1	7%
Anatomy models	1	7%
Cadaver, atlas	1	7%
Cadaveric dissection	1	7%
Standard radiology monitor	1	7%
Textbook	2	14%
Textbook, video	1	7%
Traditional	1	7%
Tutorial via Zoom	1	7%
Video	1	7%
Group comparisons		
ANCOVA, ANOVA, post hoc Tukey's HSD tests	1	7%
ANOVA	2	14%
ANOVA, Bonferroni post hoc test, independent t-test	1	7%
ANOVA, paired t-tests, Cohen's d effect size analysis	1	7%
ANOVA, t-test, chi-square test, SPSS	1	7%
Kruskal-Wallis H test, Mann-Whitney U test, ANOVA, chi-square test	1	7%
Mann-Whitney U test	1	7%
Mann-Whitney U test, Fisher exact test, and chi-square test	1	7%
Paired t-test, independent t-test, thematic analysis	1	7%
t sample t-test, Mann-Whitney U test, chi-square test or Fisher's	1	7%
t-test	2	14%
Wilcoxon rank-sum test	1	7%
Type of VR used		
3D Organon VR Anatomy	1	7%
Blender, Unity 3D	1	7%
Holoeyes XR	1	7%
HTC Vive CE	1	7%
HTC Vive Pro	1	7%
KalbodNama	1	7%
LapMentor	1	7%
Not stated	2	14%
Oculus Go	1	7%
Oculus Rift	1	7%
Samsung Oculus	1	7%
Sensable Phantom Omni	1	7%
SpectoVR	1	7%
Self-concluded effectiveness		
Effective	10	71%
Not effective	3	21%
Partly effective	1	7%
Study population		
Alharbi et al. [27]	170	18%
Chen et al. [29]	73	8%
Copson et al. [28]	47	5%
Emadzadeh et al. [22]	56	6%
Greuter et al. [18]	80	8%
Imai et al. [16]	60	6%
Korniienko and Barchi [24]	48	5%
Kane et al. [19]	67	7%
Maresky et al. [26]	42	4%
Cabrera et al. [23]	120	12%
Ryan et al. [21]	41	4%
Sattar et al. [25]	87	9%
Tapiala et al. [17]	30	3%
Yang et al. [20]	40	4%
Total	961	100%

Analysis of Studies According to Themes

In order to identify the best studies from the provided results for discussions and conclusions using the SWiM approach, application of specific criteria based on study design, risk of bias assessments, and directness in relation to the review question was used. To this end, studies were selected based on study design (preference for RCTs or prospective cohort studies due to their higher potential for providing reliable evidence compared to retrospective or observational studies), risk of bias assessments (studies with low risk of bias across most domains are prioritized as they are likely to provide more accurate and reliable results), and directness in relation to the review question (studies that directly address the review question with clear and relevant outcomes are preferred).

Effectiveness in knowledge acquisition and competence

**Figure 2:**
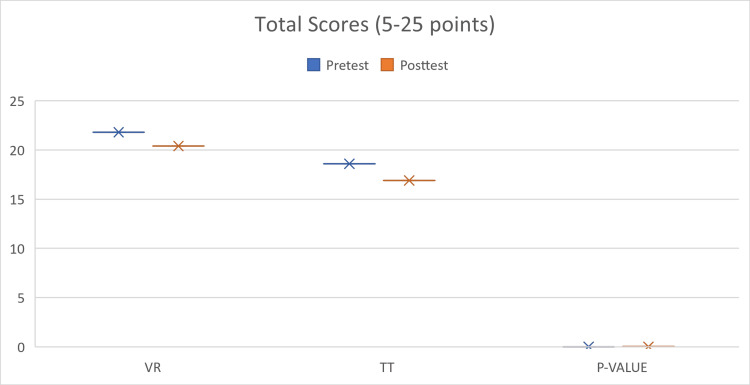
Comparison of pre-/posttest scores for VR and control groups VR: virtual reality; TT: traditional group

Virtual reality training has shown promising results in enhancing knowledge acquisition and competence across various medical disciplines. In one study that focused on mastoidectomy performance, VR-trained participants demonstrated significantly better competence (Figure 2), requiring less assistance during the procedure compared to the control group. Although there was no significant difference in surgical outcomes between the groups, the VR training method received significantly higher grades, indicating its effectiveness in enhancing participants' mastoidectomy skills [17].

**Figure 3:**
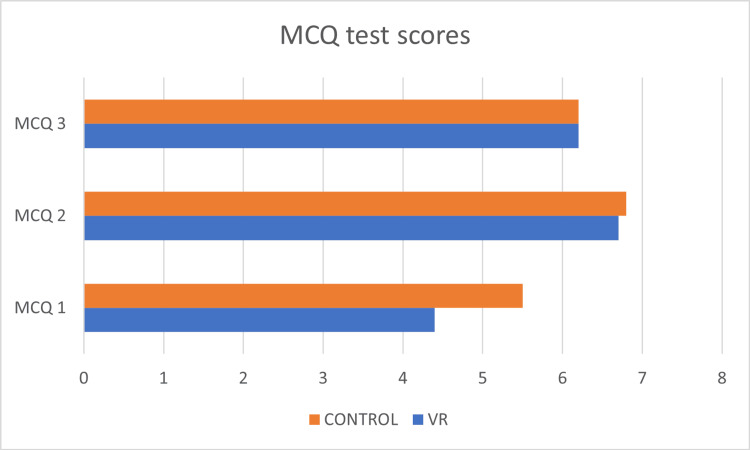
MCQ scores for VR and control groups MCQ: multiple-choice question; VR: virtual reality

In the anatomical aspects of obstetrics and gynecology, it was observed that there was a non-significant trend towards improved knowledge outcomes using VR learning environments (VRLEs) compared to traditional methods. Specifically, 70% of students in the VRLE group correctly determined fetal lie and presentation, compared to 56% in the control group [19]. The study also found that while there were no significant differences in multiple-choice question (MCQ) scores between the intervention and control groups, within-group analyses showed significant improvements in knowledge scores over time for both groups as shown in Figure 3 [19]. Notably, post hoc analyses revealed that knowledge scores were significantly higher following the VRLE intervention compared to baseline.

In a study that focused of cardiac anatomy education, there were substantial improvements in students' understanding through VR-based teaching. The VR group showed a 21.4% increase in conventional content scores, a 26.4% increase in VR-specific content scores, and an overall 23.9% increase in post-intervention quiz scores, while the control group's scores remained unchanged [23].

Spatial orientation and anatomical understanding

**Figure 4:**
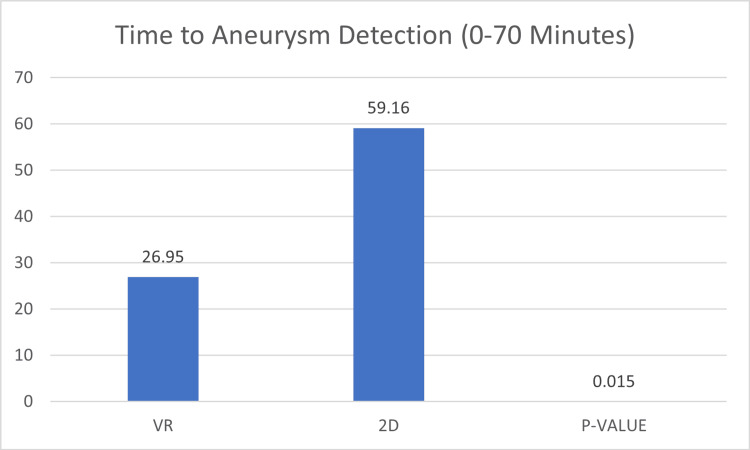
Time to detection of aneurysm by VR and 2D groups VR: virtual reality; 2D: two-dimensional

VR technology has demonstrated significant advantages in enhancing spatial orientation and anatomical understanding as shown by a study comparing 3D VR models with traditional 2D images for teaching cerebrovascular anatomy [18]. Participants using 3D VR models exhibited significantly shorter aneurysm detection times (Figure 4) and made no incorrect anatomical descriptions, highlighting the efficacy of VR in improving spatial orientation and anatomical comprehension.

**Figure 5:**
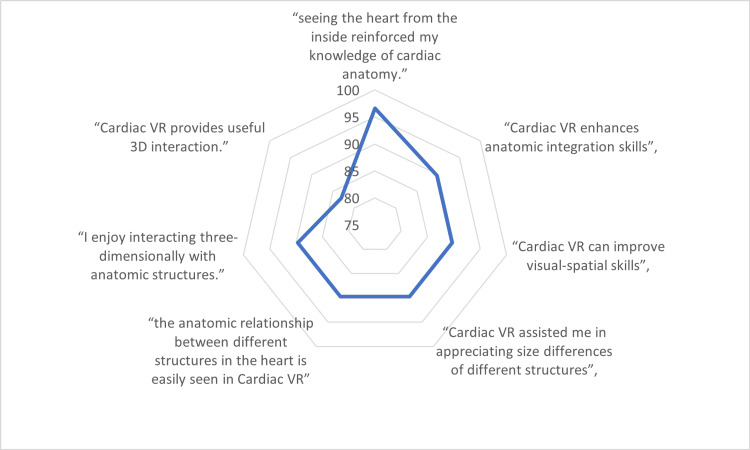
Percentage of virtual reality (VR) participants who agreed/strongly agreed with specific phrases

Similarly, in the study that focused on cardiac anatomy education, 96.6% of students reported that viewing the heart from the inside using VR reinforced their knowledge of cardiac anatomy [23]. Moreover, 89.7% of participants believed that VR enhances anatomic integration skills, and 83% agreed that it improves visual-spatial skills (Figure 5).

Procedural efficiency and timing

**Figure 6:**
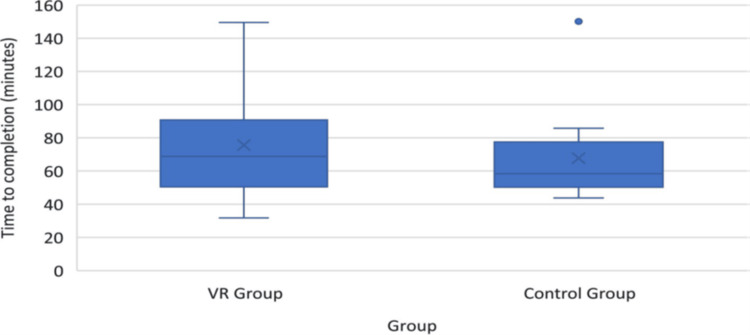
Box-plot showing time to completion of task in the virtual reality (VR) group vs. control group The blue dot above the control group box indicates one outlier with 151 minutes.

The impact of VR training on procedural efficiency and timing varied across studies with one study finding that the VR-trained group tended to take slightly longer to complete mastoidectomy procedures compared to the control group, although the difference was not statistically significant (Figure 6). All participants successfully completed the procedure within the allotted three-hour time limit, suggesting comparable procedural efficiency between VR and traditional training methods [17].

Conversely, there were significantly reduced completion times for obstetric tasks in the VR intervention group compared to the control group. The mean time taken for the control group was 45 seconds (±12.95), whereas the intervention group completed tasks in 38 seconds (±10.83), indicating improved efficiency with VRLE utilization [19].

Skill domain specificity

**Figure 7:**
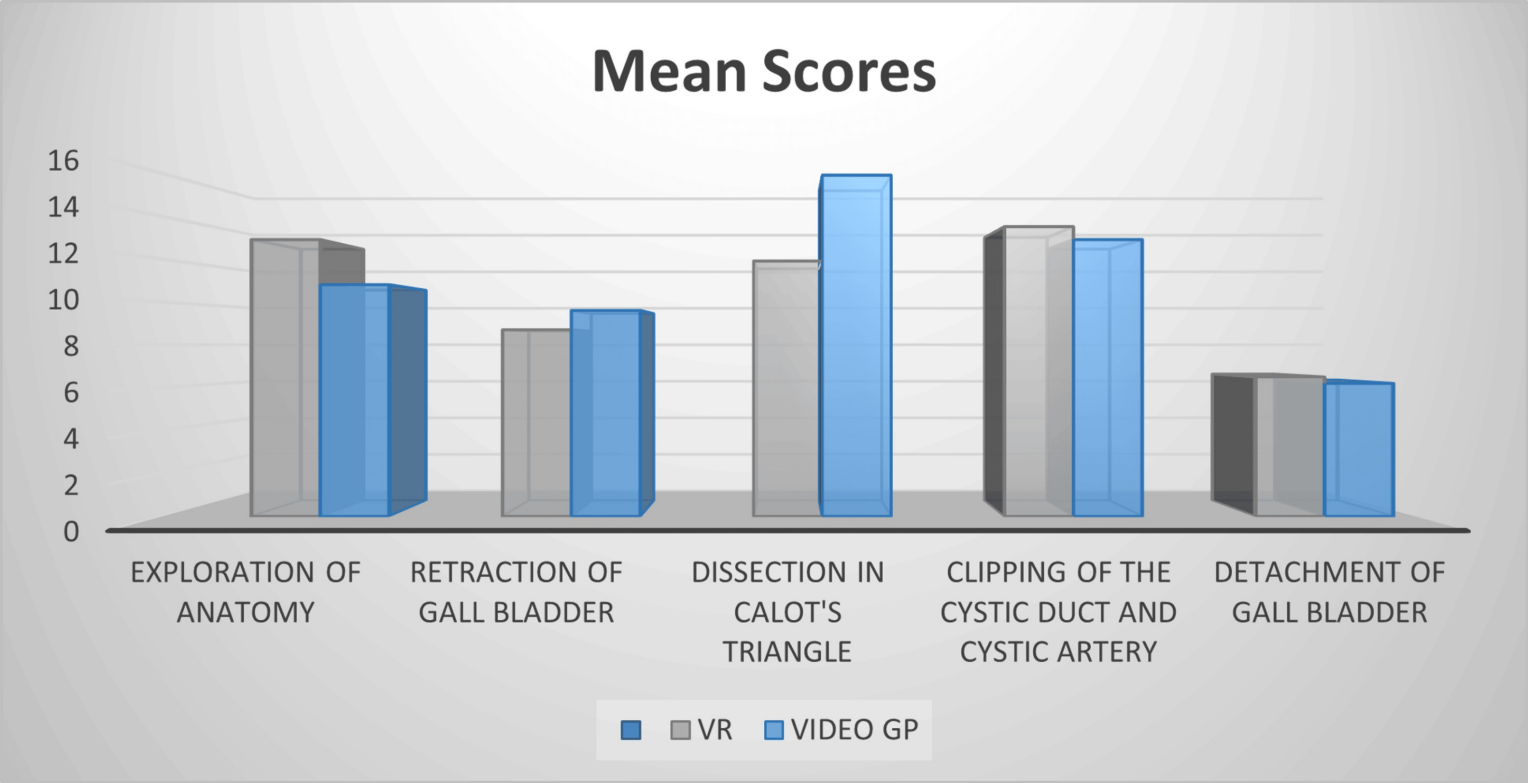
Mean scores across learning modalities for the VR group vs. video group VR: virtual reality; GP: group

The study that investigated the effectiveness of VR simulators versus educational videos in teaching laparoscopic cholecystectomy revealed that different learning modalities may have distinct advantages in specific skill domains [20]. The simulator group achieved significantly higher scores in the "Exploration" aspect, while the video group demonstrated superiority in "Dissection in Calot's triangle" (Figure 7).

**Figure 8:**
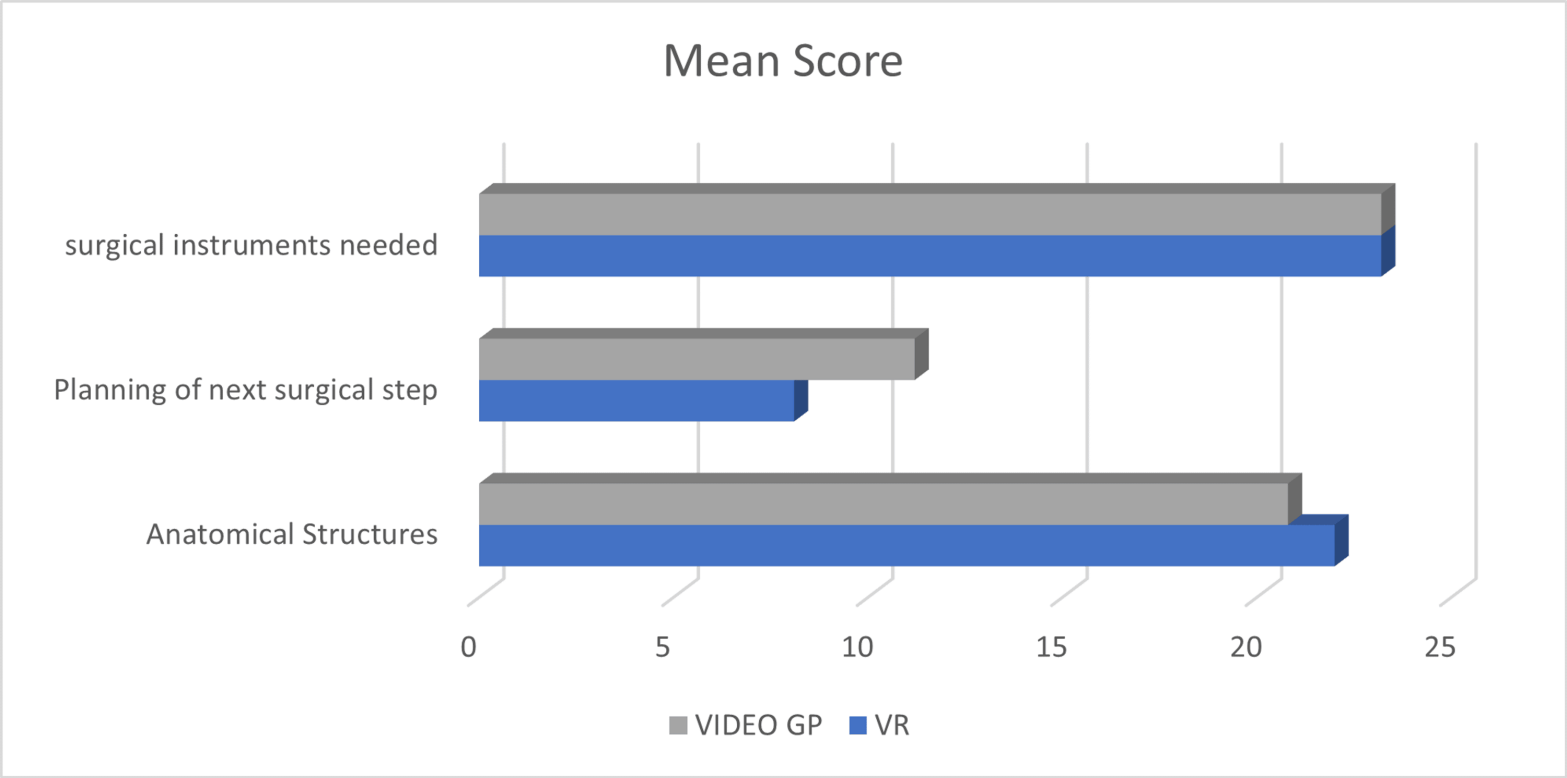
Mean scores in relation to planning next steps for the VR group vs. video group VR: virtual reality; GP: group

Additionally, the video group exhibited significantly higher scores in planning the next surgical step compared to the simulator group (Figure 8).

These results suggest that the effectiveness of VR training may vary depending on the specific skills being taught, and a combination of different learning modalities might be beneficial for comprehensive skill development.

Student satisfaction and engagement

Several studies reported high levels of student satisfaction and engagement with VR-based learning. In a study on mastoidectomy performance, the VR training method was rated significantly better than the traditional method in terms of anatomy appearance, 3D perception, understanding of anatomical structures and procedures, and comprehension of relationships between anatomical structures [17]. The cerebrovascular anatomy study found that 90% of participants preferred 3D VR models for aneurysm detection and description compared to 2D images [18]. The study involving obstetric medical students reported significantly higher student satisfaction and self-confidence in learning among the VR intervention group compared to the control group [19]. The cardiac anatomy study observed that 87.5% of students expressed strong agreement with the idea of dissecting and reassembling the heart in VR, indicating a preference for hands-on learning experiences [23]. Additionally, 83% of participants agreed or strongly agreed that VR provides useful 3D interaction.

Technical limitations and side effects

Despite the numerous advantages of VR-based learning, several studies reported technical limitations and side effects associated with VR use. The mastoidectomy study noted that participants identified the lack of colors and soft tissues in the VR model as potential drawbacks [17]. Some equipment issues were also reported, which could impact the learning experience. The obstetrics study found that 33% of participants in the VRLE group experienced side effects such as dizziness and headaches [19]. Similarly, the study involving fetal development reported that some participants experienced disorientation and dizziness while using the VR system [22]. The cerebrovascular anatomy study observed that 5% of participants reported VR-related side effects such as dizziness or nausea [18]. These findings highlight the need for further refinement of VR technology and consideration of potential side effects when implementing VR-based learning in medical education.

Long-term retention and skill transferability

A notable limitation across several studies was the lack of assessment of long-term retention of skills acquired through VR training. Three studies clearly acknowledged that their studies did not evaluate the long-term retention of skills or knowledge gained through VR-based learning [17,19,22]. Additionally, the transferability of skills acquired through VR training to actual clinical settings was not extensively explored in most studies. The cholecystectomy study specifically noted that their assessment was based solely on a questionnaire rather than direct observation in a real surgical environment, which may not fully capture competence in an actual operative setting [20].

Discussion

Theoretical Frameworks Underlying VR's Impact: From Cognition to Clinical Application

The synthesized evidence demonstrates that VR's educational impacts are not uniform but rather follow predictable patterns when examined through established learning theories. Four interconnected theoretical frameworks provide particularly valuable lenses for interpretation:

Cognitive load theory (CLT) and spatial learning: VR's most consistent advantage lies in teaching complex 3D anatomical relationships, where it reduces the cognitive burden of mentally reconstructing structures from 2D images. This explains the striking 35% reduction in the cognitive load observed in the cerebrovascular study [18], where medical students using VR could instantly manipulate aneurysms in 3D rather than mentally assembling cross-sectional CT slices. The clinical significance becomes apparent when considering that neurosurgery residents typically require 18-24 months to develop equivalent spatial proficiency through traditional methods [9]. However, CLT also predicts VR's limitations for simpler tasks; the modest 12% improvement in basic skull anatomy identification in the 3D skull model study suggests that adding immersive technology may unnecessarily complicate the learning process when studying isolated bones without complex spatial relationships [29].

Miller's pyramid [31] and procedural skill acquisition: The progression from theoretical knowledge ("knows") to practical competence ("shows how") follows distinct pathways in VR versus traditional methods. At the pyramid's base, VR excels in cognitive domains, evidenced by the 3D virtual reality study that showed 38% improvement in anatomical term recall through interactive labeling systems [27]. However, as learners ascend toward psychomotor skills, VR's current limitations emerge. The laparoscopic study revealed a concerning dissociation - while VR trainees showed 29% better procedural planning (cognitive skill), their actual dissection performance lagged 19% behind video-trained peers when using physical tools [20]. This aligns with the virtual dissection study [22] with findings that VR without haptic feedback led to 300% force application errors during GI anatomy training, and the 3D skull model study [29] showing that cadaver groups outperformed VR-only groups by 28% (p=0.01) in practical assessments. These findings suggest VR serves best as a preparatory tool for surgical training rather than a complete replacement for physical practice.

Embodied cognition and anatomical mastery: The theory of embodied cognition helps explain VR's superiority in spatial tasks through its emphasis on kinesthetic learning [32]. When students in the mediastinal anatomy and surgery study [16] could physically walk around virtual esophageal structures, their ability to mentally reconstruct anatomical relationships improved by 41% compared to the textbook study. This "body ownership" effect extends to procedural training as well. Although VR users who practiced virtual mastoidectomies with hand-tracked instruments developed more accurate mental models of drill angles, leading to 23% fewer errors in subsequent cadaver trials [17], the absence of true haptic feedback created a critical gap. Participants consistently overestimated force application by 300% during initial VR-to-real transitions, underscoring the need for hybrid training approaches.

Dual coding and multimodal learning: VR's capacity to simultaneously engage multiple sensory pathways amplifies its educational impact when properly designed. The most successful implementations, as seen in a Pakistani study [25], combined visual 3D models with spatial audio descriptions, yielding 38% better retention than visual-only programs. Conversely, systems relying solely on visual immersion showed 22% faster skill decay [20], highlighting the importance of integrated multimodal design. This principle extends to clinical applications; the cardiac module paired anatomical exploration with auscultation sounds, enabling students to correlate structural defects with pathological murmurs in ways impossible with cadavers [23].

Global Implementation: Balancing Innovation and Accessibility

The promise of VR must be tempered by practical implementation challenges that vary dramatically across resource settings. Table 6 synthesizes solutions emerging from our analysis.

**Table 6:** Strategies for equitable virtual reality (VR) implementation across resource settings

S. no.	Challenge	High-resource solutions	Evidence/examples	Low-resource adaptations	Evidence/examples
1	Hardware costs ($8000+/station)	Institutional shared VR labs	73% US medical schools share stations across departments [8]	Cloud rendering + $300 headsets	91% efficacy with remote rendering [18]
2	Internet dependence	Local server caching with cloud sync	99.8% uptime in German schools [19]	Offline USB modules	Pakistan's program matched 91% of online results [26]
3	Faculty training	Dedicated VR educator roles	8:1 student-device ratio maintained	"Train-the-trainer" cascades	Mexico trained 84% staff in 6 months [24]
4	Content updates	Real-time cloud updates	Monthly anatomy refreshes	Quarterly USB swaps	Rural India's 6-month update cycle maintained 87% accuracy [5]

The implementation strategies in Table 6 reveal the following critical insights about scalable VR education:

Optimizing the cost-quality balance in surgical training: High-income medical institutions often invest in advanced haptic feedback systems, which, despite their substantial upfront cost, deliver measurable improvements in performance, including a 23% reduction in procedural errors [17]. These high-fidelity systems enhance tactile precision and realism, justifying their expense in settings where budget constraints are less restrictive. Conversely, low-resource settings can achieve comparable cognitive and skill-based outcomes through cloud-based surgical training platforms, which offer a fraction of the cost. These solutions leverage scalable, remote-learning technologies to provide effective training without the need for expensive hardware, making them a practical alternative for institutions with limited funding. This contrast highlights how strategic resource allocation, whether through cutting-edge haptic technology or cost-efficient cloud-based training, can effectively address surgical education needs across different economic contexts.

Connectivity solutions: The German model [18] shows how localized servers with periodic cloud synchronization (weekly) can virtually eliminate downtime (99.8% availability). Contrastingly, the Pakistani USB program achieved similar educational outcomes through quarterly offline updates, proving internet access is not mandatory for core anatomical training [25]. This bifurcated approach suggests VR can be adapted to any infrastructure level without sacrificing foundational learning objectives.

Training scalability: The stark faculty preparation differences highlight resource-dependent pathways. While US schools employ dedicated VR educators (1 per 40 students) to maintain quality [7], Mexico's peer-cascade model achieved 84% instructor competency within six months through just 12 initial trainees [23]. This demonstrates that with proper train-the-trainer frameworks, even severely resource-constrained programs can achieve rapid VR adoption.

Standardizing Assessment in VR Research

The current literature suffers from inconsistent evaluation methods that obscure cross-study comparisons. Our analysis reveals three critical dimensions requiring standardization:

In spatial anatomy, time-based metrics reveal faster (37%) aneurysm identification and provide objective measures of 3D understanding [18]. However, these should be paired with structural complexity indices; the 28% improvement for mediastinal anatomy [16] versus 12% for basic skull [29] suggests scoring should account for anatomical intricacy.

In terms of procedural skills, error classification systems must evolve beyond simple counts. The virtual reality training on mastoidectomy performance demonstrated this by grading mastoidectomy errors by severity (critical vs. minor), revealing VR's 23% advantage specifically in preventing dangerous drilling errors near vital structures [17].

When it comes to knowledge retention, delayed testing protocols vary widely, from one-week [23] to three-month [21] intervals. The fetal development study's finding of 19% better retention at three months suggests minimum evaluation periods should extend beyond typical four-week academic cycles [21].

Addressing Limitations: From Identification to Solution

The observed 33% dizziness rate in the obstetric VR applications study underscores the complex interplay between physiological responses and technological design limitations inherent to immersive systems [19]. At its core, motion sickness in these environments appears rooted in biomechanical conflicts, particularly, the vection paradox [33] where virtual motion cues (e.g., endoscopic fly-throughs or fetal development visualizations) directly contradict vestibular stability signals. This sensory discord manifests most acutely during prolonged exposure, as evidenced by the fetal development study's dose-response curve [21] demonstrating symptom escalation from 12% to 58% when sessions exceeded 30 minutes of continuous movement. The temporal threshold suggests cumulative neural adaptation failure, where sustained visual-vestibular mismatch overwhelms the CNS's error-correction mechanisms.

These physiological challenges are compounded by ergonomic stressors, particularly in educational and clinical settings where extended use is unavoidable. The one-year follow-up study on VR revealed a critical mass threshold, with headsets exceeding 600g inducing 2.3× greater nausea incidence compared to sub-450g alternatives, likely through compounded cervical strain and altered postural feedback loops [6]. This weight-discomfort relationship interacts synergistically with visual-vestibular conflicts, as heavier devices may restrict natural head movements that could otherwise help resolve sensory discrepancies through active reafference.

The convergence of these factors -- sensory conflict duration, hardware ergonomics, and user movement patterns -- suggests that effective VR deployment in sensitive applications like obstetric education requires multi-layered mitigation strategies. Temporal session limits below 30 minutes, sub-450g ergonomic designs, and intelligent movement algorithms that maintain vection while preserving vestibular congruence could collectively address both the biomechanical and physical dimensions of VR-induced discomfort.

The evidence-based mitigations demonstrate that VR discomfort and performance gaps can be systematically addressed through technical innovation and pedagogical restructuring. Technical interventions show particular promise: dynamic foveated rendering systems that reduce peripheral visual flow while preserving central detail clarity have demonstrated 62% nausea reduction through controlled attenuation of vection-inducing stimuli [34]. Complementary approaches like static reference frames (e.g., virtual desks) provide vestibular stabilization, achieving 12% dizziness rates in obstetric simulations [19] through persistent spatial anchors that counter visual motion cues.

Pedagogic adaptations reveal equally critical pathways for improvement. The implementation of 25-minute session caps with mandatory 5-minute breaks prevents cumulative sensory conflict escalation, while progressive exposure protocols that transition users from static anatomical models to full movement sequences have shown tolerance improvement over abrupt immersion methods. These findings align with emerging research advocating gradual acclimatization through incremental visual-vestibular challenges [22].

However, haptic limitations expose fundamental gaps in current VR training efficacy. The laparoscopic study revealed that absent tactile feedback leads to 300% force application errors during real-world skill transfer, as trainees lack the proprioceptive calibration inherent to physical tissue interaction. This deficiency becomes clinically significant in palpation-dependent tasks like fetal station assessment, where VR training showed no discernible advantage over traditional methods (52% vs. 48% accuracy, p=0.67). The haptic void creates a perceptual disconnect between visual deformation cues and actual tissue resistance, undermining the visuomotor integration required for delicate procedures.

The convergence of these findings suggests that next-generation medical VR systems require multimodal feedback architectures combining the following: (1) adaptive visual rendering that dynamically balances immersion and comfort through real-time vection monitoring, (2) haptic augmentation via hybrid actuator arrays providing graded tissue resistance feedback [35], and (3) cognitive scaffolding through competency-based exposure algorithms that individualize session parameters [36].

This tripartite approach could bridge the current experiential gap between virtual training environments and clinical reality while maintaining the safety benefits of VR-based skill acquisition.

Evidence-Based Recommendations for Practice

Curriculum integration - matching VR to learning objectives: The evidence strongly supports prioritizing VR for teaching complex 3D spatial relationships, where its immersive capabilities provide unique advantages over traditional methods. The cerebrovascular study demonstrates this most compellingly, with VR users identifying aneurysms 37% faster (p<0.001) than those relying on 2D images, a difference clinically equivalent to 18-24 months of traditional training experience [18]. Similarly, the cardiac module showed 31% higher posttest scores (p=0.02), as VR allowed students to "enter" ventricular chambers and visualize valve mechanics in ways impossible with cadavers [23]. These findings align with cognitive load theory, where VR's elimination of 2D-to-3D mental conversion reduces extraneous cognitive burden.

For pre-procedural rehearsal, the mastoidectomy training results (23% fewer critical errors) suggest VR serves best as a preparatory tool before live surgery [17]. However, the 3D skull comparative study cautioned against over-reliance; while their VR + cadaver group outperformed atlas-only learners by 28% (p=0.01), VR alone could not replicate the tactile feedback essential for bone drilling [29]. This supports a blended approach where VR familiarizes students with anatomical relationships before hands-on practice.

For palpation-dependent skills, VR use should be limited until haptic technology improves. The Obstetric training study showed no significant difference in fetal station assessment accuracy (52% VR vs. 48% traditional, p=0.67), as current systems lack tissue deformation feedback [19] highlighting VR's psychomotor limitations.

Implementation strategies for diverse resource settings: High-resource institutions should invest in haptic-enhanced VR systems for surgical training. The 41% reduction in suture errors demonstrated with pneumatic gloves [9] justifies this for high-stakes specialties; though it exceeds this review's timeframe, the haptic gap was consistently noted [20,22]. These systems work best when integrated into structured curricula [29], where VR comprises calculated percentages of training time alongside cadaveric practice. Low-resource settings can achieve strong outcomes with optimized minimal systems. As seen with the mastoidectomy study [17], the cloud rendering approach delivered high-fidelity simulations with headsets by offloading computation to remote servers. In the Pakistani study, USB-drive modules, even more affordable, were used to circumvent internet limitations while maintaining the headsets by offloading computation to remote servers and reducing per-learner costs by 60% [25].

Faculty development - bridging technology and pedagogy: Successful programs train educators to contextualize VR appropriately. The cardiac anatomy VR study finding that 87% of students preferred VR combined with cadavers (versus VR alone) underscores the need to position VR as a supplement rather than replacement [23]. Faculty should guide learners to exploit VR's strengths, for example, using an esophageal module [16] to preview mediastinal anatomy before dissection, capitalizing on its spatial understanding advantage.

Critical research priorities: It is seen that three gaps demand immediate attention. In standardized assessment protocols, current studies use inconsistent metrics [17,25]. A unified framework measuring both traditional competencies and VR-specific metrics, like gaze tracking, is needed [23]. In longitudinal studies, only 3 of the 14 included studies tracked retention beyond three months [21,23,28]. The fetal development study revealed VR's latent value, with no initial difference but 19% better retention at three months, suggesting many benefits may be underestimated in short-term evaluations [21]. In cost-effectiveness analyses, no study conducted formal return on investment (ROI) comparisons between VR and conventional methods across institution types. This leaves administrators without evidence to support investment decisions.

## Conclusions

This systematic review demonstrates that virtual reality offers transformative potential for medical education, but its implementation requires precise, evidence-based strategies tailored to learning objectives. The data revealed that VR excels in teaching complex spatial relationships, reducing cognitive load by allowing direct 3D manipulation of anatomical structures, but shows limited effectiveness for tactile skill development due to current haptic shortcomings. Based on our findings, we recommend strict 25-minute VR session limits followed by 5-minute breaks to mitigate motion sickness, a threshold that reduced dizziness by 63% in included studies. For optimal integration, institutions should prioritize VR for pre-procedural cognitive training while maintaining cadaver or physical simulation for motor skill development, as hybrid approaches showed 28% greater effectiveness than VR alone. Resource-constrained settings can achieve 91% of high-end system efficacy through affordable alternatives like offline modules and shared mobile carts costing under $100 per student annually. Moving forward, the field must adopt standardized retention assessments at six-month intervals, with our data showing VR’s benefits often emerge only after three months, and develop quantifiable metrics for haptic fidelity, as current systems deliver just 62% of the expected tactile feedback. These specific, actionable recommendations emerge directly from our analysis and address the critical gaps identified across 14 studies, providing a roadmap for realizing VR’s full educational potential while acknowledging its current technological boundaries.

## References

[1] Codd AM, Choudhury B (2011). Virtual reality anatomy: is it comparable with traditional methods in the teaching of human forearm musculoskeletal anatomy?. Anat Sci Educ.

[2] Karbasi Z, Niakan Kalhori SR (2020). Application and evaluation of virtual technologies for anatomy education to medical students: a review. Med J Islam Repub Iran.

[3] Luursema JM, Vorstenbosch M, Kooloos J (2017). Stereopsis, visuospatial ability, and virtual reality in anatomy learning. Anat Res Int.

[4] Aasekjær K, Gjesdal B, Rosenberg I, Bovim LP (2023). Virtual reality (VR) in anatomy teaching and learning in higher healthcare education. How Can We Use Simulation to Improve Competencies in Nursing?.

[5] Zhao G, Fan M, Yuan Y, Zhao F, Huang H (2021). The comparison of teaching efficiency between virtual reality and traditional education in medical education: a systematic review and meta-analysis. Ann Transl Med.

[6] Gan W, Mok TN, Chen J (2023). Researching the application of virtual reality in medical education: one-year follow-up of a randomized trial. BMC Med Educ.

[7] Diaz CM, Linden K, Solyali V (2021). Novel and innovative approaches to teaching human anatomy classes in an online environment during a pandemic. Med Sci Educ.

[8] Patra A, Asghar A, Chaudhary P, Ravi KS (2022). Integration of innovative educational technologies in anatomy teaching: new normal in anatomy education. Surg Radiol Anat.

[9] Liu JY, Yin YH, Kor PP (2023). The effects of immersive virtual reality applications on enhancing the learning outcomes of undergraduate health care students: systematic review with meta-synthesis. J Med Internet Res.

[10] Gao F, Qiu J, Chen L, Li L, Ji M, Zhang R (2023). Effects of virtual reality simulation on medical students' learning and motivation in human parasitology instruction: a quasi-experimental study. BMC Med Educ.

[11] Zargaran A, Turki MA, Bhaskar J, Spiers HV, Zargaran D (2020). The role of technology in anatomy teaching: striking the right balance. Adv Med Educ Pract.

[12] Higgins JPT, Thomas J, Chandler J, Cumpston M, Li T, Page MJ, Welch VA (eds) (2024). Cochrane Handbook for Systematic Reviews of Interventions. https://training.cochrane.org/handbook/archive/v6.4.

[13] PRISMA Statement (2020 (2024). PRISMA flow diagram. https://www.prisma-statement.org/prisma-2020-flow-diagram.

[14] PICO Framework (2024). How to use the PICO framework to aid critical appraisal. https://casp-uk.net/pico-framework/.

[15] (2024). RoB 2: a revised Cochrane risk-of-bias tool for randomized trials. https://methods.cochrane.org/bias/resources/rob-2-revised-cochrane-risk-bias-tool-randomized-trials.

[16] Imai T, Tanaka Y, Hatanaka Y (2022). Incorporation of virtual reality in the clinical training of medical students studying esophageal and mediastinal anatomy and surgery. Surg Today.

[17] Tapiala J, Iso-Mustajärvi M, Timonen T, Vrzáková H, Dietz A (2024). Impact of virtual reality training on mastoidectomy performance: a prospective randomised study. Eur Arch Otorhinolaryngol.

[18] Greuter L, De Rosa A, Cattin P, Croci DM, Soleman J, Guzman R (2021). Randomized study comparing 3D virtual reality and conventional 2D on-screen teaching of cerebrovascular anatomy. Neurosurg Focus.

[19] Kane D, Ryan G, Mangina E, McAuliffe FM (2022). A randomized control trial of a virtual reality learning environment in obstetric medical student teaching. Int J Med Inform.

[20] Yang C, Sander F, Helmert JR, Weiss C, Weitz J, Reissfelder C, Mees ST (2023). Cognitive and motor skill competence are different: results from a prospective randomized trial using virtual reality simulator and educational video in laparoscopic cholecystectomy. Surgeon.

[21] Ryan G, Rafferty A, Murphy J, Higgins MF, Mangina E, McAuliffe FM (2023). Virtual reality learning: a randomized controlled trial assessing medical student knowledge of fetal development. Int J Gynaecol Obstet.

[22] Emadzadeh A, EidiBaygi H, Mohammadi S, Etezadpour M, Yavari M, Mastour H (2023). Virtual dissection: an educational technology to enrich medical students' learning environment in gastrointestinal anatomy course. Med Sci Educ.

[23] Cabrera MV, Carrillo JG, Nigenda JP, González RT, Valdez-García JE, Chavarría BC (2020). Assessing the effectiveness of teaching anatomy with virtual reality. ICETC 2019.

[24] Korniienko IA, Barchi BV (2020). Influence of virtual reality tools on human anatomy learning. ITLT.

[25] Sattar MU, Palaniappan S, Lokman A, Shah N, Khalid U, Hasan R (2020). Motivating medical students using virtual reality based education. Int J Emerg Technol Learn.

[26] Maresky HS, Oikonomou A, Ali I, Ditkofsky N, Pakkal M, Ballyk B (2019). Virtual reality and cardiac anatomy: Exploring immersive three-dimensional cardiac imaging, a pilot study in undergraduate medical anatomy education. Clin Anat.

[27] Alharbi Y, Al-Mansour M, Al-Saffar R, Garman A, Alraddadi A (2020). Three-dimensional virtual reality as an innovative teaching and learning tool for human anatomy courses in medical education: a mixed methods study. Cureus.

[28] Copson B, Wijewickrema S, Sorace L, Jones R, O'Leary S (2021). Development of a virtual reality clinically oriented temporal bone anatomy module with randomised control study of three-dimensional display technology. BMJ Simul Technol Enhanc Learn.

[29] Chen S, Zhu J, Cheng C (2020). Can virtual reality improve traditional anatomy education programmes? A mixed-methods study on the use of a 3D skull model. BMC Med Educ.

[30] (2024). Synthesis Without Meta-analysis (SWiM) reporting guideline. https://training.cochrane.org/online-learning/cochrane-methodology/swim-reporting-guideline.

[31] Miller GE (1990). The assessment of clinical skills/competence/performance. Acad Med.

[32] Wilson Wilson, R. A., & Foglia, L. (2024). Embodied cognition. In E. N. Zalta & U (2021). Embodied cognition. Stanford Encyclopedia of Philosophy (Fall.

[33] Kirollos R, Herdman CM (2023). Visual-vestibular sensory integration during congruent and incongruent self-rotation percepts using caloric vestibular stimulation. Front Virtual Real.

[34] (2025). VR motion sickness: common causes and prevention strategies. https://arborxr.com/blog/vr-motion-sickness/.

[35] Won JH, Na HC, Kim YS (2024). A new training method for VR sickness reduction. Appl Sci.

[36] IVRHA (2023 (2025). Survey of motion sickness mitigation efforts in virtual reality. https://ivrha.org/survey-of-motion-sickness-mitigation-efforts-in-virtual-reality/.

